# Regulated Polyploidy in Halophilic Archaea

**DOI:** 10.1371/journal.pone.0000092

**Published:** 2006-12-20

**Authors:** Sebastian Breuert, Thorsten Allers, Gabi Spohn, Jörg Soppa

**Affiliations:** 1 Goethe University, Institute for Molecular Biosciences, Frankfurt, Germany; 2 Institute of Genetics, University of Nottingham, Queen's Medical Centre, United Kingdom; Duke University, United States of America

## Abstract

Polyploidy is common in higher eukaryotes, especially in plants, but it is generally assumed that most prokaryotes contain a single copy of a circular chromosome and are therefore monoploid. We have used two independent methods to determine the genome copy number in halophilic archaea, 1) cell lysis in agarose blocks and Southern blot analysis, and 2) Real-Time quantitative PCR. Fast growing *H. salinarum* cells contain on average about 25 copies of the chromosome in exponential phase, and their ploidy is downregulated to 15 copies in early stationary phase. The chromosome copy number is identical in cultures with a twofold lower growth rate, in contrast to the results reported for several other prokaryotic species. Of three additional replicons of *H. salinarum*, two have a low copy number that is not growth-phase regulated, while one replicon even shows a higher degree of growth phase-dependent regulation than the main replicon. The genome copy number of *H. volcanii* is similarly high during exponential phase (on average 18 copies/cell), and it is also downregulated (to 10 copies) as the cells enter stationary phase. The variation of genome copy numbers in the population was addressed by fluorescence microscopy and by FACS analysis. These methods allowed us to verify the growth phase-dependent regulation of ploidy in *H. salinarum*, and they revealed that there is a wide variation in genome copy numbers in individual cells that is much larger in exponential than in stationary phase. Our results indicate that polyploidy might be more widespread in archaea (or even prokaryotes in general) than previously assumed. Moreover, the presence of so many genome copies in a prokaryote raises questions about the evolutionary significance of this strategy.

## Introduction

Polyploidy, the existence of multiple copies of the normal set of chromosomes, is widely distributed in eukaryotes. Polyploids are common among fish and amphibian and very common among plants. It has even been proposed that the diploid vertebrate genomes were derived by reduction from polyploids [1]. The advantages and disadvantages of being polyploid have recently been reviewed [2], [3]. In short, the advantages of polyploidy are heterosis (hybrid vigour, an increased perfomance of the allopolyploid compared with the inbred parents), loss of self-incompatibility leading to the gain of asexual reproduction, and gene redundancy. The specific advantages of gene redundancy may be 1) a reduced chance that deleterious recessive mutations become homozygous, and 2) the potential for gene diversification and the acquisition of new functions. Disadvantages of being polyploid are 1) a higher frequency of mitotic or meiotic problems leading to aneuploidy and 2) epigenetic instability. Several of these points like heterosis apply only to eukaryotic species with sexual reproduction, while others like gene redundancy are relevant for archaea, bacteria and eukaryotes.

In contrast to eukaryotes, prokaryotes are commonly thought to contain one copy of a circular chromosome. The gram-negative bacterium *Escherichia coli* contains one complete chromosome when its generation time is longer than the time required for replication and segregation of the chromosome. Unlike eukaryotes, it does not have a G2 phase, and cell division is initiated soon after replication has been finished [4]. Under optimal laboratory conditions, the generation time of *E. coli* can become less than the replication time, and a new round of replication is initiated before termination of the previous round. The cells become merodiploid or merooligoploid for the origin-proximal genes [4], and if replication reinitiation is prevented with rifampicin, end up with 2, 4 or 8 chromosomes [5].

The best-studied gram-positive bacterium, *Bacillus subtilis*, also harbors one copy of the chromosome [6]. For *B. subtilis* and subsequently also for other bacterial species it was found that the chromosome is not distributed randomly in the cell. Replication takes place at a fixed site in the middle of the cell, and the newly replicated regions of the chromosome are immediately transported toward the cell poles [6], [7]. Thus, the DNA-polymerases and the replication forks are somewhat stationary and the DNA is highly mobile (“factory model of replication”) [7].

Several bacteria are known to be polypoid. The best known example is the radioresistant species *Deinococcus radiodurans*, which harbours about 8 chromosomal copies [8]. Another example is *Azotobacter vinelandii* that has been reported to contain up to 80 chromosome copies [9]. However, this is only seen in fast growing cultures, while cultures grown in synthetic medium are not polyploid [10]. A few other examples exist, but are thought to be exceptions from the rule that bacteria are monoploid.

Genome copiy numbers were also determined for several species from the third domain of life, the archaea. Two *Sulfolobus* species and *Archaeoglobus fulgidus* were found to have an extensive G2 phase; thus they contain two copies during most of the cell cycle, and one copy prior to replication [11]–[13]. *Methanothermobacter thermoautotrophicus* has no G2 phase, but grows in filaments that contain several nucleoids, each of which contains a single chromosome. [14]. *Methanococcus jannaschii* contains multiple copies of the chromosome, but has a very relaxed cell cycle control. The division plane is not at mid-cell, and there is no even distribution of the copies to the daughter cells [15].

The genome copy number of the haloarchaea, had not been systematically investigated until now. It has been reported that *H. cutirubrum* may contain 6–10 genome copies [16], and we have previously shown that in *H. salinarum*, intracellular localization of the DNA is highly regulated in the course of the cell cycle [17]. This prompted us to study the ploidy of *H. salinarum* and a second haloarchaeal model species, *H. volcanii*, during growth under different conditions. Surprisingly, it was found that both species are polyploid, and that the genome copy number is downregulated as cells enter stationary phase.

## Results

### *Halobacterium salinarum* has multiple copies of the chromosome

It was previously reported that *H. cutirubrum* contains six to ten copies of the chromosome [16]. This was calculated after quantitation of the total DNA content and of the cell density of a culture aliquot. We applied a similar approach to determine the ploidy of *H. salinarum* (see Materials and Methods), using *E. coli* grown in synthetic medium as a control (under these conditions, *E coli* is known to contain on average slightly more than one copy of the chromosome). We confirmed that the genome copy number of *H. salinarum* is significantly higher than that of *E. coli*, similar to *H. cutirubrum* (data not shown). However, the variation in our results was somewhat high, especially if different methods of DNA isolation were used. We therefore wanted to confirm the results using independent methods with internal standardization.

**Figure 1:**
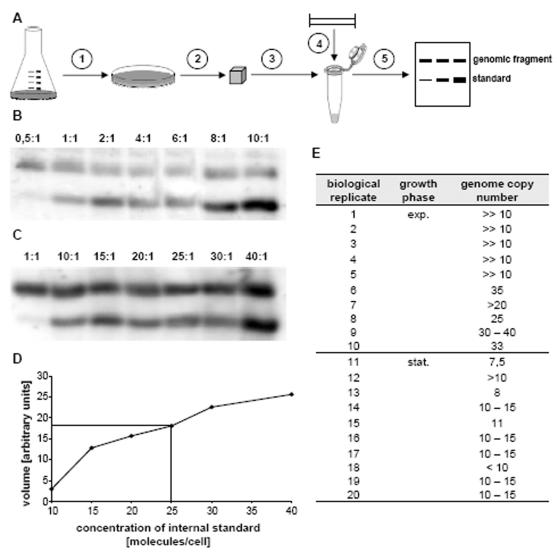
A. Overview of the method. A culture of known cell density is embedded in low melting point agarose (step 1), agarose blocks with a defined number of cells are prepared, the cells are lysed and protein is digested (step 2). The blocks are melted and a restriction enzyme (step 3) as well as an internal standard (step 4) are added. After overnight digestion, DNA fragments are size fractionated by electrophoresis and a Southern blot is performed (step 5). A 1 kbp genomic fragment near the replication origin and the 0.9 kbp internal standard are both visualized with a single probe. Multiple aliquots containing different standard concentration are used for quantitation. B. Quantitation of the genome copy number of exponential cells. After gel electrophoresis and southern blotting, a genomic fragment (upper band) and different concentrations of an internal standard (lower band) were visualized with the same probe (step 5 in A). C. Quantitation of the genome copy number of stationary phase cells. After gel electrophoresis and Southern blotting, a genomic fragment (upper band) and different concentrations of an internal standard (lower band) were visualized with the same probe (step 5 in A). D. An example of a standard curve generated after quantitation of the bands shown in B. and C. The horizontal and vertical lines show the usage of the standard curve to determine the genome copy number in the biological replicate No. 9 (see E.). E. Summary of the results of the independent cultures that were used to determine the genome copy number with the “agarose block method”. In the first five experiments, the standard curve ranged from 0.5 molecules/cell to 10 molecules/cell (as in B.). The signal of exponential phase cells was much higher than the highest signal of the standard curve and could not be quantitated. Therefore the standard curve was chosen to range from 1 molecules/cell to 40 molecules/cell (as in C.) in subsequent experiments.

An overview of the first method is given in Fig. 1A. In short, a culture of known cell density is embedded in low melting point agarose and agarose blocks with a defined number of cells are prepared. A genomic fragment of about 1 kbp near the origin is visualized by Southern blot analysis. The same probe is used for the simultaneous detection of a 0.9 kbp PCR fragment that was added as an internal standard. A standard curve is generated by the variation of the ratio of the molecules of internal standard per cell (see Material and Methods). The method allows the absolute determination of the intracellular copy number of specific DNA fragments and is not influenced by the presence of additional replicons.

Fig. 1 B shows the result for an exponential phase culture, Fig. 1C shows an example of a stationary phase culture, and Fig. 1D shows one representative standard curve used for quantitation. The results of 20 independent biological replicates are summarized in Fig. 1 E. It was found that stationary phase cells contain slightly more than 10 copies of the chromosome, while the ploidy of exponentially growing cells is considerably higher with around 30 copies/cell. Thus the polypoidy was verified and the results indicated that the ploidy of *H. salinarum* is growth phase-regulated. However, the “agarose block method” is time-consuming, inhibiting processing of large sample numbers, and the variability is somewhat greater than desired.

### The ploidy of *H. salinarum* is growth-phase regulated

**Figure 2:**
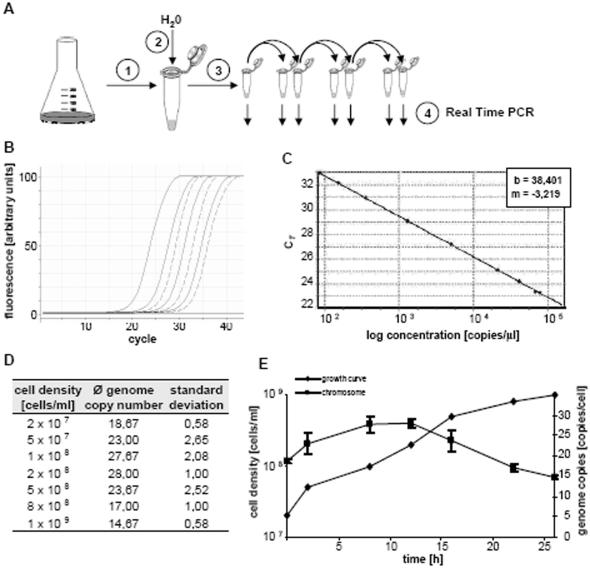
A. Overview of the method. In short, a defined number of cells was harvested and lysed (steps 1 and 2). Serial dilutions of the cell lysate (step 3) were used as templates in quantitative Real Time PCR assays (step 4). Quantitation was performed by comparison with an external and an internal standard curve (Materials and Methods). B. Selected real time PCR results. The fluorescence intensity curves from four standard dilutions (solid lines) and three sample dilutions (broken lines) are shown. In both cases serial tenfold dilutions of the templates were used. Note the identical slope of all curves and the equidistance of the curves of a dilution series, which is very close to the theoretical offset of 3.32 cycles per tenfold dilution. In addition to the selected reactions shown here, each experiment included more standards and more sample dilutions (filling the gap between tenfold dilutions) as well as a sample dilutions including a dilutions series of the standard added as internal control of PCR efficiency. C. A standard curve including nine standard concentrations distributed over three orders of magnitude. D. Average genome copy number values of three independent cultures and their standard deviation. E. Growth phase-dependent regulation of ploidy of H. salinarum. E is a graphical representation of the results tabulated in D.

A second method for genome copy number quantitation is shown in Fig. 2A, and was used to study growth phase dependent regulation of ploidy. In short, a defined number of cells was harvested and a specific genomic fragment was determined by Real Time PCR. Absolute quantitation was enabled by using external and internal standardization (see Material and Methods). This novel method combined several advantages: it is very fast and precise, extremely sensitive, and includes only a small number of steps. Fig. 2B shows the Real Time PCR results using a dilution series of the cell lysate and of the standard as templates; all curves have virtually ideal slopes and offsets. A standard curve (Fig. 2C) shows the high precision of DNA quantitation by Real Time PCR.

The method was used to determine the ploidy of *H. salinarum* throughout its growth. Three independent experiments were performed, and the results are tabulated in Fig. 2D and shown graphically in Fig. 2E. Cells in the mid-exponential growth phase contain about 25 copies of the chromosome, and this value drops to 15 copies in early stationary phase. The two independent methods used are in good agreement, and show that replication and cell division can be regulated independently in *H. salinarum*; in late exponential phase one round of cell division occurs while replication has already ceased.

### Ploidy of *H. salinrum* at different growth rates

**Figure 3:**
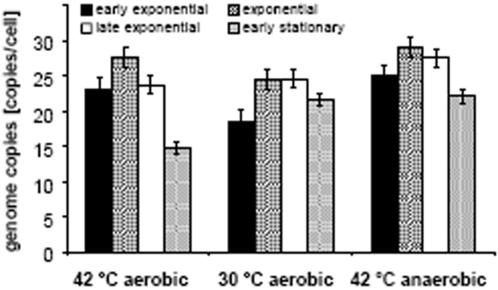
H. salinarum was grown by aerobic respiration at 42oC and 30 oC and by arginine fermentation at 42oC. The doubling times were 4 hours, 8 hours and 8 hours, respectively. For each condition three independent cultures were used. Aliquots representing early exponential phase (2–3×108 cells/ml), mid-exponential phase (4–5×108 cells/ml), late exponential phase (8–9×108 cells/ml) and early stationary phase (1–2×109 cells/ml) were used to determine the genome copy number using the Real Time PCR method. Average values of the three biological replicates and their standard deviation are shown.

The doubling time of *H. salinarum*in grown aerobically in complex medium at 42°C is about 4 hours. This is longer than the presumed replication time, given a genome size of 2.57 Mbp, and therefore meropolyploidy of origin proximal regions similar to fast growing *E. coli* is not expected. Nevertheless, we investigated whether the growth rate has an effect on ploidy, as had been reported for fast-growing *E. coli* and additional bacterial species (see Discussion). Two different conditions were used that lower the growth rate by about a factor of two, 1) cultures were grown at 30°C instead of 42°C, and 2) they were grown anaerobically by arginine fermentation instead of aerobic respiration. In both cases, the doubling time is around 8 hours. Samples were taken at four time points representing early, mid- and late exponential as well as early stationary phase. We found no reduction in ploidy in exponential phase at lower growth rates, where cells contained at least 25 genome copies under all three conditions (Fig. 3). The only difference is seen in early stationary phase, where a smaller reduction of ploidy was seen in slow-growing cells (8 hour doubling time) than in fast-growing cells (4 hour doubling time). As a consequence, stationary phase cells have a varying DNA content that depends on their history and thus they seem to have a “molecular memory” of what conditions they had experienced before they became stationary.

### Ploidy of different *H. salinarum* replicons and its regulation

**Figure 4:**
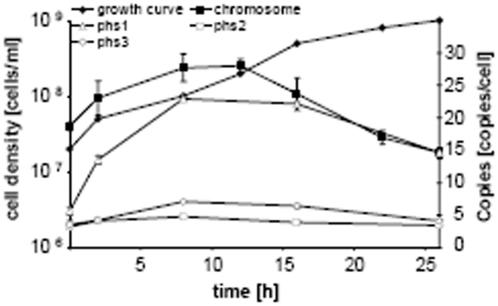
Three independent cultures were used to determine the copy numbers of the chromosome and three additional replicons, i.e. pHS1 to pHS3, using the Real Time PCR method. One of the growth curves, the average replicon copy number per cell and the standard deviations are shown.

*H. salinarum* contains several replicons [http://www.halolex.de/], and the results above pertain to the largest replicon (chromosome). We set out to quantitate the copy numbers of the additional replicons termed pHS1 to pHS4. Since the smallest replicon pHS4 is only present in the sequenced strain (DSM 671) but not in the strain used for this study (DSM 670), the analysis was restricted to pHS1 to pHS3. Real Time PCR assays for the three replicons were established, and the ploidy throughought the culture growth was quantitated in parallel with the chromosome (Fig. 4). Surprisingly the copy numbers of two of the plasmids, pHS 2 and pHS3, are much lower than that of the chromosome. Furthermore, their copy number is not regulated and is around five from early exponential phase to stationary phase. The ploidy of the third plasmid, pHS1, is regulated similar to that of the chromosome, but it has a much lower copy number in early exponential phase. Therefore at least two different mechanisms of replicon copy number regulation exist in *H. salinarum*.

### Ploidy of *Haloferax volcanii* and its regulation

**Figure 5:**
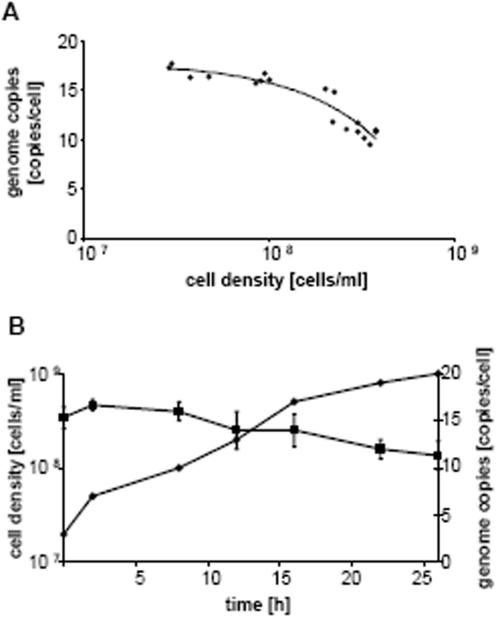
Determination of genome copy number using the agarose block method. Different aliquots from one culture were used to inoculate several new cultures that were grown overnight. Throughouht the next day aliquots were withdrawn, the cell density was determined with a counting chamber and the ploidy with the agarose block method. For each aliquot, the cell density was plotted against the genome copy number, and a trend line was calculated. B. Determination of the genome copy number using the Real Time PCR method. Three independent cultures were used to determine the genome copy number. A selected growth curve, the average ploidy values and their standard deviation are shown.

To investigate whether the polyploidy is restricted to *H. salinarum* or might be more widely distributed in haloarchaea, *H.* volcanii was chosen as a second example. It belongs to another genus and differs considerable from *H. salinarum* in morphology, metabolism and behavior. The growth phase-dependent ploidy of *H. volcanii* was quantitated using the two different methods described above. Initial experiments were performed with the “agarose block method” and show that *H. volcanii* is also polyploid (Fig. 5A). Similar to *H. salinarum*, there is a negative correlation between the cell density and the chromosomal copy number, indicating growth phase-dependent copy number regulation.

The genome copy number was also determined by the quantitative PCR method, which yielded near-identical results. *H. volcanii* has almost 20 genome copies in exponential growth phase, and around 12 copies in stationary phase. Thus, it has a slightly lower genome copy number and a somewhat lower degree of growth phase-dependent copy number regulation than *H. salinarum*.

### Distribution of genome copy numbers in single cells of the population

The two methods for genome copy number determination yielded information about the average values in the population, but gave no information about the variation of genome copy number in individual cells.

As a first approach to address the ploidy of single cells, confocal laser scanning microscopy (CSLM) was used. *H. salinarum* cells of the mid-exponential as well as of the stationary growth phase were fixed with formaldehyde and the DNA was stained with the fluorescence dye Hoechst 33342. As a control that the physiological state had been preserved, it was verified that all different intracellular DNA localization patters could be found in the cells from the exponentially growing culture, i.e. distributed DNA, one focus in the middle of the cell, three foci, and two foci at the cell poles [17]. Furthermore it was verified that the localization patterns that are specific for ongoing replication were absent from cells from stationary phase cultures (data not shown). 50 cells of each culture were chosen randomly and the fluorescence signal was quantitated. The average values were 163 a.u. (standard deviation 75) for cells from the exponential phase and 83 a.u. (s.d. 18) for cells from the stationary growth phase. Thus the growth phase-dependent regulation of the genome copy number could also be verified on the single cell level. The high standard deviation especially in the exponential phase provided a first indication that the genome copy number might be variable in the population. However, the method was not suitable to quantitate the variation of genome copy numbers in the population since it is not exact enough and it is difficult to characterize sufficient cells to address the question in a statistically significant manner.

**Figure 6:**
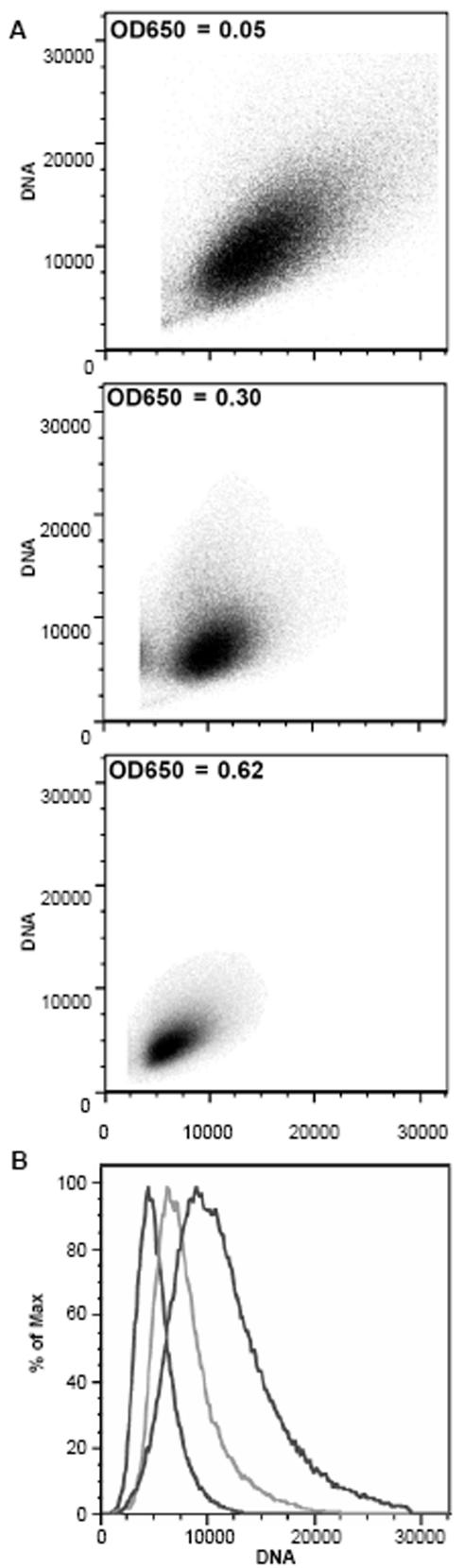
A. The forward light scatter as a measure of cell size is plotted against the fluorescence of the DNA-specific dye acridine orange as a measure of the DNA content. Aliquots representing early and middle exponential phase and early stationary phase (top to bottom) were analyzed, and the optical densities are indicated. B. The fluorescence as a measure of DNA content is plotted against the fraction of the population exhibiting a specific fluorescence. The three curves from left to right represent the three aliquots shown in A from top to bottom.

As an alternative, we used a “fluorescence activated cell sorter” (FACS) to determine the variation of the DNA content in a high number of cells. Again, the growth phase-dependent regulation of ploidy could be verified on the single cell level (Fig. 6). Thus, the “agarose block method” and the “Real Time PCR” method on the one hand and the CLSM and the FACS analyses on the other hand yield identical results, while they have two important differences: 1) the former methods determine the average content in the population, while the latter methods address single cells, and 2) the former methods quantitate a specific DNA sequence, while the latter determine the overall DNA content with a fluorescent dye. The FACS analysis is the only method that allows to address the variation of DNA content in a large population of cells. Fig. 6 shows that the variation is much larger in exponentially growing than in stationary phase cells, the latter being much more uniform in DNA content as well as in size.

## Discussion

Two very different haloarchaea model species [18] were chosen for the quantitation of the genome copy number. They are dissimilar concerning e.g. cell morphology, optimal salt concentration, motility and taxis, utilizable electron donors and receptors, and the presence of haloarchaeal retinal proteins and gas vesicles. However, both species were found to be highly polyploid and to downregulate the number of genome copies as cells enter stationary phase. This indicates that polyploidy might be widespread in the different genera of halobacteriaceae and might even be more widespread in archaea as previously anticipated. Until now *M. jannaschii* was the only archaeal species that had been found to contain multiple copies of the chromosome [15]. However, the number of species that were found to contain one chromosome in the G1 phase and two chromosomes in the G2 phase of the cell cycle is very limited (*Sulfolobus, Archaeoglobus, Methanothermobacter*) [12]–[14].

The situation is similar for bacteria. It is generally assumed that most bacterial species are monoploid and contain a single copy of a circular chromosome. But also in the bacterial domain of life, the number of genome copies has only be quantitated for a limited number of species. *E. coli* has been characterized very thoroughly and it is clear that slow-growing cells contain one chromosomal copy while fast growing cells are merooligoploid for origin-proximal regions, e.g. cells growing with at a doubling time of 24 minutes contain, on average, 6.54 origins and 1.94 termini [4]. A small number of other bacteria have also been shown to be monoploid, i.e. *Bacillus subtilis* and *Comamonas testosteroni* [6], [19].

We have shown that *H. salinarum* is not only highly polyploid, but also possesses a strict cell cycle control. Upon addition of the DNA polymerase inhibitor aphidicolin, cells stop dividing immediately [17], although the high genome copy number should allow several divisions without the risk of generating anucleate cells. *H. volcanii* is also highly polyploid, and we have isolated temperature sensitive mutants that stop dividing after a shift to the restrictive temperature (unpublished data). These data indicate that polyploidy is not just the result of the loss of proper genome copy number control, but that it offers selective advantages that led to its development in evolution.

Several bacterial species have been reported to contain multiple copies of the chromosome. Probably the best known example is the radioresistant species *Deinococcus radiodurans*. It contains at least four genome copies and no monoploid stage could be detected at different growth rates [8]. The human pathogen *Neisseria gonorrhoeae* contains on average three genome copies, suggesting that nascent *Neisseria* cells contain two genome copies, which are replicated in a concerted fashion to generate a cell with four genome copies [20]. As both species are coccal organisms, a linkage between morphology and polyploidy has been postulated [20]. However, species of other morphologies have also been found to be polyploid. *Desulfovibrio gigas* contains about nine genome equivalents in ammonia-limited chemostat cultures and about 17 genome copies in fast growing batch cultures [21]. *Borrelia hermsii*, a spirochete that causes relapsing fever, harbors about 16 genome copies if it is grown in mice but only 1/4 to 1/2 of this number if it is grown axenically in batch culture [22]. The case of *Azotobacter vinelandii* is controversial. A gene hybridization study concluded that this species harbors as many as 80 genome copies [9], [23]. In contrast, a genetic approach came to the conclusion that “*A. vinelandii* is not a polyploid bacterium” [24]. One line of evidence was that “heterozygotic transconjugants and transformants are unstable and segregate into homozygotic colonies even in the absence of selection”. A subsequent study by the same group used flow cytometry and came to the conclusion that the genetic approach had let to erroneous results (see below) and that *A. vinelandii* is indeed polyploid. Fast-growing cells in the early exponential phase contained about the same number of chromosomes than *E. coli*, but the number of genome copies highly increased to as many as 100 in late exponential and in early stationary phase. However, it was also found that in synthetic medium at lower growth rate the genome copy number did not change and the authors concluded that “the polyploidy of *A. vinelandii* may not exist outside of the laboratory” [10].

Taken together, there are more characterized “exceptions” from the rule that bacteria are monoploid than species that have been proven to follow the rule. Polyploidy might be more widespread in prokaryotes than presently anticipated, and it will be interesting to quantitate the genome copy number in more archaeal and bacterial species. No common pattern has emerged from the characterized archaeal and bacterial species that are polypoloid. For example, the range of genome copy numbers is extremely large (from 4 to greater 100), cells may have a higher genome copy number in exponential phase (haloarchaea) or in stationary phase (*Azotobacter*), and the genome copy number may change with growth rate (*Desulfovibrio, E. coli*) or is independent from growth rate (*Halobacterium*).

It has been proposed that a selective advantage of polyploidy in prokaryotes could be a higher resistance to DNA damaging conditions, especially those that induce DNA double strand breaks. The radioresistant species *D. radiodurans* can survive X-ray dosages that lead on average to more than 150 double strand breaks per chromosome [25]. However, a study where the genome copy number of *D. radiodurans* was altered by growth in different media found no correlation between ploidy and resistance to gamma or UV radiation [26]. *H. salinarum* is also extremely resistant to X-ray irradiation. The D_10_ values (10% survival) are 10 kGy for *D. radiodurans* and 5 kGy for *H. salinarum* [27], [28]. For comparison, the D_10_ value of *E. coli* is 0.25 kGy [29]. However, *H. volcanii*, which has a similar genome copy number to *H. salinarum*, is not particularly radioresistant (D_10_ = 1 kGy, unpublished data).

It is possible that polypolidy has evolved in response to desiccation, which is known to induce DNA double strand breaks. The hypersaline environments of haloarchaea are characterized by high temperatures and a high intensity of sun light, therefore the cells are always in danger of desiccation. *H. salinarum* was shown to be extremely desiccation resistant; about 25% of the cells remained viable after the exposure to high vacuum (10^−6^ Pa) for 20 days [28].

One selective advantage of polyploidy might be that many genome copies allow the accumulation of mutations in different alleles of any gene without losing the wildtype allele. This might be important in situations where growth of the cells is compromised and alleles coding for e.g. enzymes with an altered substrate specificity or affinity might restore the ability to grow.

While different alleles at the same locus might exist under specific conditions, genetic evidence suggests that this is not the case under normal growth conditions. For example, we have found it easy to isolate haloarchaeal mutants in different metabolic pathways, such as bacteriorhodopsin-mediated phototrophic growth, arginine fermentation, nitrate respiration, and cell cycle progression [30, 31, unpublished data]. Only two rounds of selection with an enrichment factor of about 10^3^ are necessary, which argues against random segregation of a mutated gene from 15 to 25 wildtype alleles [30]. A second line of evidence is the ease of replacing wildtype genes of *H. salinarum* and *H. volcanii* with deletion mutations, indicating efficient homologous recombination [32]–[35]. Thirdly, *H. volcanii* possesses an efficient genetic exchange system. Mutants can be crossed and the segregation of the different chromosomes that had been combined into one cell by cell fusion can be followed by phenotypic markers. After a limited number of generations, the alleles had segregated in the absence of selection, and cells showed only one of the two parental phenotypes [36].

These results indicates that *H. salinarum* and* H. volcanii* possess an efficient gene conversion mechanism that guarantees that all genome copies in growing cells are equalized. It is clear that gene conversion exists in archaea and bacteria, but it was only thought to operate on multiple copies of a gene that are situated on the same chromosome, e.g. ribosomal RNA genes [e.g. 37, 38]. However, it was recently demonstrated that gene conversion is used to eliminate deleterious mutations in the genomes of plastids, which are polyploid [39]. It will be interesting to study whether gene conversion also operates to equalize alleles on different chromosomes of polyploid archaea and bacteria.

An additional selective advantage of polyploidy might be that it allows to globally control gene expression by gene dosage regulation. The degree of regulation is nearly twofold for the main replicon and nearly fivefold for pHS1. Under non-saturating concentrations of regulatory proteins, which is often physiologically relevant, the occupancies of regulator binding sites in the DNA change with altered replicon copy numbers. Depending on the regulatory mechanism this can result in repression or induction of gene expression. In addition, the presence of different regulons with different ploidy and different regulary patterns allows *H. salinarum* to include different gene dosages in a single cell, that vary by a factor of 5. However, if the gene dosage would influence the expression level of some or many genes, it would also mean that the expression of theses genes is not uniform in exponentially growing *H. salinarum* cells, since the genome content of single cells was found to be very variable (Fig. 6). Clearly, methods for quantitation of gene expression levels at the single cell level are needed to clarify this question. In stationary phase, the genome content of the cells within the population is much more uniform. However, it depends on the history of the cells, and thus stationary phase cells have a “molecular memory” of the conditions they were exposed to during their growth phase. In another project, the transcript level of a specific gene in stationary phase cells was also found to vary in dependence of their history (unpublished data).

### Coda

*H. salinarum* and *H. volcanii*, representing two very different haloarchaeal genera, were both found to be highly polyploid. The genome copy number is higher than in any other prokaryote, with the exception of fast-growing *A. vinelandii* under laboratory conditions. Four different methods have shown that the ploidy level is regulated and stationary phase cells habor a smaller number of chromosomes than exponentially growing cells. The copy numbers of different replicons were quantitated, two different species were investigated, the average population as well as single cells have been studied, and different growth conditions and temperatures were used. Thus, this is one of the most thorough reports describing the genome copy number in prokaryotes. The results warrant a closer look at the distribution of polyploidy in archaea and bacteria.

## Materials and Methods

### Archaeal and bacterial strains and growth conditions

*H. salinarum* was obtained from the German culture collection (http://www.dsmz.de/; strain No. DSM670). It was grown in complex medium by aerobic respiration or by arginine fermentation as described previously [31], [40]. *H. volcanii* strain H26 was constructed and grown in complex medium as described [33]. The *H. volcanii* strain WR340 was obtained from Moshe Mevarech (Tel Aviv University, Israel) and grown in complex medium [41]. *Escherichia coli* strain Xl1-blue MRF' was obtained from Stratagene (Leiden, Netherlands) and grown in M9 synthetic medium [42]. *E. coli* strain MG1655 [43] was grown in LB broth or 56/2 salts medium with 0.4% glucose or 0.2% glycerol.

### Quantitation of ploidy using isolated genomic DNA

Two different aproaches were used to isolate genomic DNA from *E. coli* and *H. salinarum*. The first approach was to use the DNeasy tissue kit according to the instructions of the manufacturer (Qiagen, Hilden, Germany). The second approach was to precipitate DNA with ethanol after cell lysis and protein digestion. For both species, the cell density was determined using a Neubauer counting chamber. For the isolation of DNA from *E. coli*, 15 ml culture was harvested by centrifugation (15 min., 4000 g, 4°C). The pellet was suspended in 3.6 ml lysis buffer (10 mM Tris/HCl, pH 7.2, 1 mM EDTA, 100 mM NaCl, 0.05% SDS). 30 µl lysozyme solution (100 mg/ml) and 400 µl proteinase K solution (1 mg/ml) were added and it was incubated for 4 hours at 37°C. The solution was extracted three times with an equal volume of phenol and once with ether. DNA was pelleted by the addition of 1/10 vol. sodium acetate solution (3 M, pH 5.2) and 2.5 vol. ethanol. After centrifugation (20 min., 12 000 g, 4°C), the pellet was solved in TE (10 mM Tris/HCl, pH 7.2, 1 mM EDTA). For the isolation of DNA from *H. salinarum*, 15 ml of culture was pelleted (see above) and the cells were suspended in 300 µl of basal salt solution (medium without carbon source). 2.5 ml lysis buffer (60 mM EDTA, 0.25% sodium sarconisate, 60 mM Tris/HCl, pH 8) were added and it was mixed until lysis was complete. 400 µl proteinase K solution were added and it was incubated for 4 hours at 37°C. Phenol extraction and ethanol precipitation was performed as described above.

The DNA concentration was determined photometrically. To calculate the genome equivalents, genome sizes of 4.64 Mbp and 2.57 Mbp were used for *E. coli* and *H. salinarum* [44], [45], that correspond to 5.1 fg/genome and 2.8 fg/genome for the two species.

### Quantitation of ploidy using the “agarose block method”

The cell density was determined with a Neubauer counting chamber. Cells from culture aliquots were harvested by centrifugation (15 min., 6000 g, room temperature) and resuspended in 1/10^th^ volume of basal salts [31]. 4 ml of cell suspension were quickly mixed with 4 ml of a 2% (w/v) solution of low melting point agarose (agarose type VII with low gelling temperature, Sigma, Steinheim, Germany). A 1 ml aliquot is transferred to an eppendorf tube and the rest is poured into a Petri dish. The weight of the 1 ml aliquot is determined and–together with the known cell density of the suspension–allows to calculate the number of cells per unit weight. After the agarose had solidified in the Petri dish, small ararose blocks of 4×4×4 mm were generated with a self-constructed device. The weight of the agarose blocks were determined and the number of included cells was calculated. The blocks were washed twice for 30 minutes in 50 volumes TE (10 mM Tris/HCl, pH 7.2, 1 mM EDTA). After that, they were incubated overnight at 37°C in 20 volumes lysis buffer (60 mM EDTA, 0.25% (w/v) sodium sarconisate, 100 µg/ml proteinase K, 60 mM Tris/HCl, pH 8.0). They were washed three times for 20 minutes in 10 volumes TE+1 mM PMSF, then they were washed three times for 30 minutes in 10 volumes TE. Subsequently they were transferred invidivually into pcr tubes. The agarose was molten at 80°C and then cooled to 37°C. The desired ratio of internal standard molecules per cell was added. As an internal standard, a pcr fragment was used that was generated with the primers SB_002_ST and SB_003_ST (Table 1) and genomic DNA of *H. salinarum* as a template. It was purified using a preparative agarose gel and the Quiaex II gel extraction kit (Qiagen, Hilden, Germany). It represents a genomic region close to the presumed replication origin of *H. salinarum*. 4.5 µl of the restriction enzyme Sal I (10 U/µl) and 6.5 µl of 10× Sal I buffer were added. Sal I does not cleave the internal standard, but it generates a 1 kbp fragment from the genomic DNA that fully includes the sequence of the internal standard. After incubation at 37°C for three hours they samples were shortly heated to 50°C and transferred to a 1.4% (w/v) agarose gel. Gel electrophoresis, transfer to a nylon membrane, hybridization, washing and detection with a digoxigenin-labelled probe were performed as previously described [46]. The probe was constructed using the internal standard pcr fragment as a template (primers see Table 1). Thus the probe equally recognizes the 900 bp internal standard pcr fragment and the 1 kbp genomic SalI fragment. Hybridization signals were visualized by using an anti-DIG-alkaline phosphatase conjugate and the chemoluminscence substrate CDP-star according to the instructions of the manufacturer (Roche, Mannheim, Germany) and exposing the nylon membranes to an X-ray film. The film was scanned and the signals were quantitated with the software ImageJ 1.32 (NIH, Bethesda, USA). For each band the background was determined using a field of the same surface area directly above and below the band. The average background value was subtracted from the signal of the band. The signals from the internal standards were used to construct a standard curve, which was used to determine the genome copy number using the average signal from the 1 kbp genomic fragment.

**Table 1:**
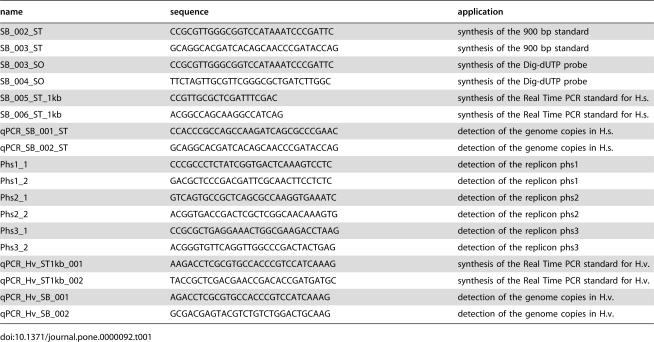
Primer used for determination of the genome copy numbers

name	sequence	application
SB_002_ST	CCGCGTTGGGCGGTCCATAAATCCCGATTC	synthesis of the 900 bp standard
SB_003_ST	GCAGGCACGATCACAGCAACCCGATACCAG	synthesis of the 900 bp standard
SB_003_SO	CCGCGTTGGGCGGTCCATAAATCCCGATTC	synthesis of the Dig-dUTP probe
SB_004_SO	TTCTAGTTGCGTTCGGGCGCTGATCTTGGC	synthesis of the Dig-dUTP probe
SB_005_ST_1kb	CCGTTGCGCTCGATTTCGAC	synthesis of the Real Time PCR standard for H.s.
SB_006_ST_1kb	ACGGCCAGCAAGGCCATCAG	synthesis of the Real Time PCR standard for H.s.
qPCR_SB_001_ST	CCACCCGCCAGCCAAGATCAGCGCCCGAAC	detection of the genome copies in H.s.
qPCR_SB_002_ST	GCAGGCACGATCACAGCAACCCGATACCAG	detection of the genome copies in H.s.
Phs1_1	CCCGCCCTCTATCGGTGACTCAAAGTCCTC	detection of the replicon phs1
Phs1_2	GACGCTCCCGACGATTCGCAACTTCCTCTC	detection of the replicon phs1
Phs2_1	GTCAGTGCCGCTCAGCGCCAAGGTGAAATC	detection of the replicon phs2
Phs2_2	ACGGTGACCGACTCGCTCGGCAACAAAGTG	detection of the replicon phs2
Phs3_1	CCGCGCTGAGGAAACTGGCGAAGACCTAAG	detection of the replicon phs3
Phs3_2	ACGGGTGTTCAGGTTGGCCCGACTACTGAG	detection of the replicon phs3
qPCR_Hv_ST1kb_001	AAGACCTCGCGTGCCACCCGTCCATCAAAG	synthesis of the Real Time PCR standard for H.v.
qPCR_Hv_ST1kb_002	TACCGCTCGACGAACCGACACCGATGATGC	synthesis of the Real Time PCR standard for H.v.
qPCR_Hv_SB_001	AGACCTCGCGTGCCACCCGTCCATCAAAG	detection of the genome copies in H.v.
qPCR_Hv_SB_002	GCGACGAGTACGTCTGTCTGGACTGCAAG	detection of the genome copies in H.v.

### Quantitation of ploidy using the “Real Time PCR method”

#### Overview

The rationale of the “Real Time PCR method” is to harvest haloarchaeal cells and lyse them completely by an osmotic shock in low salt solution. Dilutions of the cell lysates are used directly as templates in Real Time PCR assays. Quantitation of the genome copy number is achieved by comparing the results with a dilution series of a pcr product of known concentration that is used as a standard. To ensure that the pcr amplification with the genomic templates has the same efficiency as the amplifications with the pcr product, a third series of Real Time PCR assays was used. A dilution series of the pcr standard was added to the cell lysates and it was verified that the efficiency of standard amplifiction was identical in the absence and presence of cell lysate.

#### Real time pcr method

*H. salinarum* or *H. volcanii* were grown in complex medium as described [31], [33]. It was taken care that they were growing for at least 25 generations in exponential phase before they were used for genome copy number determinations. At the times indicated for each experiment the cell density was determined using a Neubauer counting chamber, and 2 ml aliquot of the cell culture was harvested by centrifugation (5 min., 3800 g, room temperature). The pellet was suspended in 1 ml of basal salt solution [31], [33]. 200 µl of the cell suspension was added to 1.8 ml of a. bid., resulting in rapid cell lysis. Serial dilutions of the cell lysate were generated in a. bid., and 5 µl aliquots were directly used as template for Real Time PCR assays.

For the quantitation of the genome copy number by Real Time PCR a standard was needed. A 1 kbp pcr product was generated using genomic DNA of *H. salinarum* as a template (primers see Table 1). It was purified by preparative agarose gel electrophoresis, eluted with the Quiaex II gel extraction kit (Quiagen, Hilden, Germany), and the concentration was determined photometrically. A series of dilutions were prepared that contained defined numbers of standard molecules, and 5 µl aliquots were used as templates for Real Time PCR. To ensure that pcr efficiencies of the cell lysate dilution series and the standard dilution series were identical, a third series of samples were prepared. A standard dilution series was added to one of the cell lysate samples as an internal control.

The Real Time PCR assays contained in a 25 µl reaction volume 5 µl of template (cell lysate, standard, or cell lysate with added standard), 1 µM of primer qPCR_SB_001 and primer qPCR_SB_002, 2× qPCR Master Mix (Finnezymes OY, Espoo, Finnland), and 17.1% (v/v) glycerol. The master mix contained *Thermus brockianus* DNA polymerase, SYBR GREEN I, dNTPs, MgCl_2_, and buffer (concentrations not released by the manufacturer). The pcr reaction conditions were 10 min. at 96°C, 40 cycles of 30 sec. 96°C, 45 sec. 68°C, 80 sec. 72°C, and an additional incubation of 5 min at 72°C. The Real Time PCRs were performed in the “Rotor Gene 3000” (Corbett Research, Melbourne, Australia). 72 samples could be analyzed simultaneously. At the end of the pcr, the probes were heated to 96°C and the melting point of each sample was determined. Data analysis was performed using the software “Rotor Gene 6.35” (Corbett Research). For each sample the number of cycles was determined until its fluorescence intensity reached a threshold determined by the software (C_t_ value). The C_t_ values of the standards were used to construct a standard curve (compare Fig. 2C) that was used to quantitate the genome copy numbers in the cell lysates and to check the pcr efficiencies in the lysates including internal standards.

#### Optimization of the method

As the method was established, different steps were optimized to generate the protocol outlined above. Different cell lysis methods was compared, and it turned out the results were most reproducible if the cells were first harvested and resuspended in basal salts before they were lysed with a. bid. The cell density of the culture and of the cell suspension after centrifugation and resuspension were determined and it was revealed that the loss of cells was about 7% (this was be included in the calculation). The calculated melting points of the primers should be at least 75°C to allow an annealing temperature of 68°C. Different pcr fragment lengths were tested (100 bp, 350 bp, and 500 bp), and the 350 bp fragment turned out to be amplified most reproducibly without any artificial byproducts. The addition of glycerol turned out to be important because it abolished degradation of the genomic DNA during Real Time PCR that was otherwise observed. Real time pcr kits of different suppliers were tested, and the kit “DyNAmo qPCR kit 400L-Fi” from Finnezymes turned out to be the best.

### Fluorescence microscopy

500 µl aliquots of cultures of the mid-exponential and the early stationary growth phase were harvested by centrifugation (5 min., 3800 g, room temperature). The pellet was resuspended in 500 µl of fixing buffer (4% (v/v) formaldehyde, 0.1 M sodium cacodylate, 4.2 M NaCl, 220 mM MgCl_2_, 40 mM MgSO_4_, pH 7.0) and incubated for 10 minutes at 42°C. The cells were pelleted again under identical conditions, resuspended in 500 µl of washing solution (20 mM MgCl_2_, 20 mM MgSO_4_, 10 mM Tris/HCl, pH 7.5) and incubated for 10 minutes at 42°C. The dye Hoechst 33342 was added to a final concentration of 15 µg/ml and it was incubated for 20 minutes at 42°C. Subsequently the cell suspension was layered onto agarose-covered microscope slides. Microscopic pictures were obtained using a confocal laser scanning microscope (Leica TCS SP5) and TIFF images were analyzed using the software Imaris 4.1.1 (Leica).

### FACS analysis

*Halobacterium salinarum* DSM670 was grown in complex medium as described [31], and the growth phase was determined by optical density. A sample corresponding to about 1×10^8^ cells was harvested by centrifugation (6 minutes, 3800 g, room temperature) and resuspended in 450 µl of basal salt solution. 50 µl of acridine orange solution (Sigma, 0.1 mg/ml in basal salt solution) was added to a final concentration of 10 µg/ml, and samples were analyzed promptly. Sample analysis was performed with an Apogee A40 mini flow cytometer (equipped with a 50 mW 488 nm solid state laser (Coherent). A 510–580 nm bandpass filter was used to detect acridine orange fluorescence due to DNA binding. A PMT voltage of 415 V was used for forward light scatter and 590 V used for emission between 510 and 580 nm (gain = 1). The light scatter signal was used as the trigger parameter. Latex beads of a uniform size and fluorescence were used for calibration and adjustment of the flow cytometer.
